# Innovation in Weight Loss Programs: A 3-Dimensional Virtual-World Approach

**DOI:** 10.2196/jmir.2254

**Published:** 2012-09-20

**Authors:** Jeanne D Johnston, Anne P Massey, Celeste A DeVaneaux

**Affiliations:** ^1^Department of KinesiologyIndiana UniversityBloomington, INUnited States; ^2^Operations and Decision Technologies DepartmentIndiana UniversityBloomington, INUnited States; ^3^Club One IslandSan Francisco, CAUnited States

**Keywords:** Virtual world, obesity, weight loss programs, Internet technology, behavior change

## Abstract

**Background:**

The rising trend in obesity calls for innovative weight loss programs. While behavioral-based face-to-face programs have proven to be the most effective, they are expensive and often inaccessible. Internet or Web-based weight loss programs have expanded reach but may lack qualities critical to weight loss and maintenance such as human interaction, social support, and engagement. In contrast to Web technologies, virtual reality technologies offer unique affordances as a behavioral intervention by directly supporting engagement and active learning.

**Objective:**

To explore the effectiveness of a virtual-world weight loss program relative to weight loss and behavior change.

**Methods:**

We collected data from overweight people (N = 54) participating in a face-to-face or a virtual-world weight loss program. Weight, body mass index (BMI), percentage weight change, and health behaviors (ie, weight loss self-efficacy, physical activity self-efficacy, self-reported physical activity, and fruit and vegetable consumption) were assessed before and after the 12-week program. Repeated measures analysis was used to detect differences between groups and across time.

**Results:**

A total of 54 participants with a BMI of 32 (SD 6.05) kg/m^2 ^enrolled in the study, with a 13% dropout rate for each group (virtual world group: 5/38; face-to-face group: 3/24). Both groups lost a significant amount of weight (virtual world: 3.9 kg, *P *< .001; face-to-face: 2.8 kg, *P *= .002); however, no significant differences between groups were detected (*P *= .29). Compared with baseline, the virtual-world group lost an average of 4.2%, with 33% (11/33) of the participants losing a clinically significant (≥5%) amount of baseline weight. The face-to-face group lost an average of 3.0% of their baseline weight, with 29% (6/21) losing a clinically significant amount. We detected a significant group × time interaction for moderate (*P *= .006) and vigorous physical activity (*P *= .008), physical activity self-efficacy (*P *= .04), fruit and vegetable consumption (*P *= .007), and weight loss self-efficacy (*P *< .001). Post hoc paired *t *tests indicated significant improvements across *all *of the variables for the virtual-world group.

**Conclusions:**

Overall, these results offer positive early evidence that a virtual-world-based weight loss program can be as effective as a face-to-face one relative to biometric changes. In addition, our results suggest that a virtual world may be a *more effective *platform to influence meaningful behavioral changes and improve self-efficacy.

## Introduction

Recent national survey data indicate that 35.7% of the US population is obese and 68.8% overweight [1]. Obesity can reduce quality of life and increase the risk for many serious chronic diseases and premature death. As little as a 5%-10% reduction in weight can diminish the risk for diseases such as coronary heart disease and type 2 diabetes [2]. While there are a variety of means to achieve weight loss, one approach is a formal weight loss program. Traditionally, these programs have been delivered via face-to-face programming either in the community or in a commercial or clinical setting. They typically emphasize lesser rather than dramatic changes via weekly small group meetings over a multiweek (16-26 weeks) period. On average, these programs produce a weight loss of 0.4 to 0.5 kg per week with an overall loss of 7%-10% [3,4].

Over the last decade or so, Internet or Web-based programming has emerged [5-9]. This is not surprising given that, as of 2012, the global Internet penetration rate was reported as 32.7%, with North America leading all geographic regions at 78.6% [10]. American adults use the Web for an increasing array of activities, including over 80% who report seeking health-related information [11]. In the context of weight loss, Web offerings address many of the challenges of face-to-face programming, including location-neutral access and often lower cost [12-14]. And, for people who find face-to-face programming intimidating, online programs offer some degree of, if not complete, anonymity. However, results of Web-based weight loss interventions have been highly variable and often with small effect sizes [7,9,15-17]. This may be due to the fact that most are intended to inform (eg, they are educational or diagnostic) and are not necessarily designed to elicit behavior change. In addition, two recent reviews have emphasized the importance of including a behavioral change component when using technology-based programs to influence weight loss [8,15].

The importance of behavioral change in face-to-face interventions is well established [18-20]. Specifically, behavioral-based programs aim to change habits though the use of self-monitoring techniques, cognitive restructuring, and social support [3,12]. The premise is that changing daily habits and behaviors (eg, diet and physical activity) is necessary to induce weight loss and longer-term maintenance [21-23]. To induce change, the design of interventions may be informed by an understanding of effective learning environments, including providing plenty of social interaction; making the environment intrinsically rewarding; and ensuring engagement through active learning [24]. For example, social support helps create a learning environment, which provides a context for positive behaviors and the development of self-efficacy [25]. Furthermore, weight loss interventions may benefit from advances in understanding how people learn and the reality that participatory media (eg, mobile and online games, and social media) are increasingly important components of the lives of many people.

Given all this, behavioral change may be advanced by integrating an increased understanding of behavioral learning with innovative technologies. However, despite helping to overcome face-to-face challenges, current Web-based interventions are typically missing some important aspect of human (social) interaction *and *often fall well short of delivering truly engaging experiences—both are key elements of effective learning environments. In contrast to Web technologies, 3-dimensional (3D) virtual reality technologies more directly support engagement and active learning [26]. Based on education and behavioral theories, this should lead to improved outcomes. Early evidence suggests, for example, that simulated experiences via virtual reality can help develop self-efficacy [27]. To date, however, research has largely involved costly and sophisticated virtual reality (eg, head-mounted displays, cave automatic virtual environments) [28]. Interest has recently expanded to 3D virtual worlds (virtual worlds) [7,29-32].

### Description of Virtual Worlds

Virtual worlds share many of the strengths of virtual reality technologies, particularly the rendering of 3D spaces. However, they are more accessible to users (via an Internet-connected personal computer) and thus may offer a way to extend the reach of programs to obese and overweight individuals. Virtual worlds also possess affordances that differentiate them (to varying degrees) from virtual reality and Web technologies. Specifically, virtual worlds are *persistent *(continuing to exist even when users are not logged in) and, as multiuser spaces, they support *social interactivity *[7,33]. Since individuals can be influenced by others, social interactivity may promote positive behaviors via emotional (eg, encouragement) or informational (eg, advice or knowledge) support [25,34,35]. In principle, virtual-world users experience a sense of *presence*—the feeling of being there in the virtual place rather than in the physical space where one’s body is really located [36]. The notion of *being *there is also enhanced by the possibility of *doing *there [37], a necessary condition for active learning. Participants act within the virtual world through the use of an *avatar*—a digital representation of self, typically customizable so a user can portray an actual or desired self-image [38].

Importantly, the ability to customize one’s avatar self and use it to interact with others allows for a new way to assert one’s embodied subjectivity. Early research suggests that it is avatar identification that matters so significantly for identity and behavior modification. The effect of one’s experience in a virtual world on one’s being in the physical world has been termed the Proteus effect [39]. This effect results in users transferring expectations or understandings of their avatar’s behavior to their own real-world behavior [40]. This phenomenon may have similarities to how behavior is learned from others in the real world who act as role models, as posited by social cognitive theory [23]; may aid self-efficacy; and, in turn, may lead to improvement in health behaviors [41,42]. To illustrate, Fox et al [40] conducted a study in which participants were exposed (via head-mounted displays) to either a virtual representation of self running on a treadmill, a virtual representation of an other running, or a virtual representation of self loitering. Follow-up surveys 24 hours after the experiment revealed that participants in the virtual representation of self running condition reported significantly higher levels of physical activity than those in the other two conditions.

Based on these ideas, the purpose of the present study was to examine the effectiveness of a virtual-world-based weight loss intervention program in achieving weight loss, behavioral change, and self-efficacy.

## Methods

### Context of Study

Club One Island is an interactive weight loss community delivered via Linden Lab’s Second Life [43], an online virtual world. An island is essentially virtual land that can be built on and customized. Club One Island provides participants with a professional team, education, and specialized tools to help them overcome individual barriers to weight loss. Access to educational components and specialized tools is available 24 hours a day, 7 days a week, along with virtual world and email access to instructors. Club One Island can be described relative to two main, interrelated elements: *island *and *program design*. The design of both was informed by social cognitive theory, concepts from gaming, prior research on weight loss interventions; and emerging research on avatar identification.

**Figure 1:**
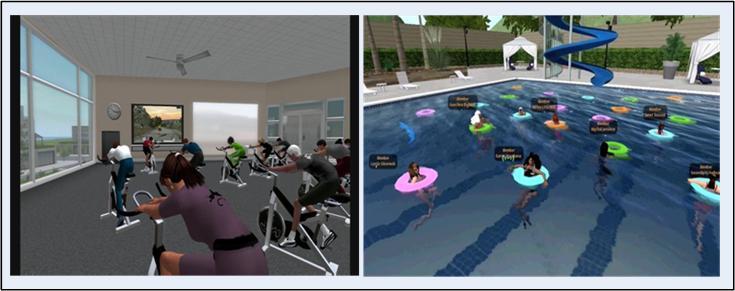
Examples of program activities in Club One Island.

**Figure 2:**
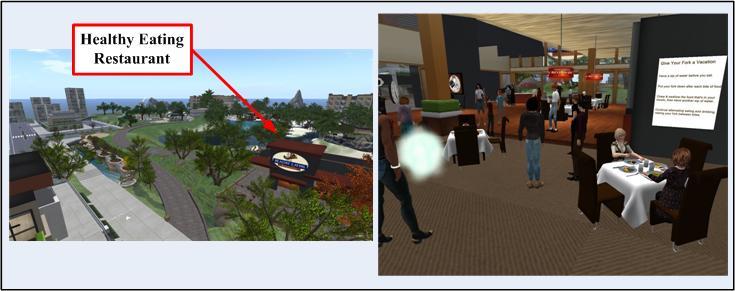
Design of the Club One Island virtual weight loss community.

First, Club One Island was designed to be visually and functionally engaging. It offers highly interactive 3D spaces (eg, a restaurant, a Mini-Mart convenience store, and an encouragement room), creative educational tools (eg, a nutritional jeopardy game and a fire pit illustrating how the body uses food as fuel), over 30 movement activities (eg, bikes and bike paths, surfing, exercise balls, lap swim, basketball, weight lifting, yoga, dancing, and rock climbing), and numerous healthy habits tools (eg, tracking charts). All elements are intended to engage participants in social networking, play, and learning. For example, the restaurant area was designed for use as a practice area for nutrition planning. It has an interactive menu that displays a full selection of items that a participant might encounter during a restaurant outing. The menu works on a stoplight (red, yellow, green) model, and participants are asked to choose what they believe are the healthiest choices. The menu responds to their selection with the color appropriate to their selection and an explanation as to why this menu item was ranked at that particular color, as well as ways to make the item healthier. Topics discussed are hidden calories, eating out, and portion control. The restaurant area also includes an ice cream counter, dining tables, a dessert bar, and a bar area. In addition, the social support classes were designed to provide an environment within which the participant could reflect on the past week’s activities and social bonds. To this end, social support classes took place in the pool, at the camp fire on the beach at sunset, and while performing yoga poses. Overall, Club One Island was designed to provide an environment that closely mirrors the physical world. By setting up learning situations that incorporate practicing new behaviors (eg, throwing away 3D food, addressing the “food pushers” and nonsupportive people in their lives, and doing any physical activity in public), Club One Island is intended to help participants overcome their fears related to weight loss. Figure 1 and Figure 2 show examples of available activities and participants using the restaurant area.

Second, the weight loss program itself was designed to move participants from a diet and exercise cycle of weight loss and gain to a view that they are on a *healthy life path *that does not have a stop and end date but is maintainable for the rest of their lives. The 12-week program was delivered to cohorts of 15–20 participants for a total of 48 instructor hours. Each week, four 1-hour classes (*Nutrition, Movement, Healthy Habits*, and *Support Group*) were led by certified fitness, nutrition, and support professionals. Each week addressed a common theme (eg, emotion as related to eating) across all 4 classes.

In the program, participants chose how their avatar looked (actual or desired) and made modifications over time, as wanted. In addition, the Nutrition, Movement, and Healthy Habits classes were all designed in such a way that participants were always moving. For example, participants (ie, their avatars) in Movement sessions engaged in 1 to 4 different activities, ranging from roller skating to surf boarding to riding bicycles, to swimming and more. Similarly, during the Nutrition and Healthy Habits classes, participants spent 90% of their class time in (virtual) standing and moving positions. When proceeding from one activity to the next, they would run, bike, roller skate, etc, to get there. These program design elements were intended to encourage avatar identification, leading to the transfer of virtual behaviors to the physical world. Multimedia Appendix 1 provides an overview of Club One Island’s virtual spaces and participant activities.

### Participants

We conducted a study of Club One Island’s weight loss program, comparing it with a face-to-face program similar in structure and content offered in a commercial setting (owned by Club One Inc., San Francisco, CA, USA). Specifically, this program included instructor-led weekly educational sessions on nutrition, movement, and habit change, as well as a social support group meeting. Program participants were also able to use club equipment and facilities during normal business hours.

Club One Island’s virtual-world program involved 38 participants recruited via print and online media. Participants had to be over the age of 18 years, with a body mass index (BMI) of 25 kg/m^2 ^or greater, and have access to an Internet-connected computer. They were told they were helping to assess a new program and, as such, it was free. Of the 38 enrollees, 1 was a current Club One member, 9 were members of competing clubs, and 28 did not belong to any health club. Since the comparison face-to-face group had to attend a real facility, we recruited a convenience sample (via email and newsletters) from Club One’s member base. Enrollees had similar age and BMI requirements, leading to a 24-person face-to-face cohort. Across all enrollees, they had on average previously tried 2 other weight loss programs. Prior to the start of the virtual-world program, participants received technical training and support (eg, computer setup and navigating the island).

### Measures

Data were collected by trained professionals at a Club One facility and provided to the researcher in de-identified form. Both objective and self-report measures were captured at baseline (preprogram) and within 1 week of program completion. Objective data (height, weight, and BMI) were recorded using standard techniques. We calculated the percentage change in baseline weight; a reduction of 5% or more of baseline weight results in clinically significant health benefits [44]. The baseline and postsurveys captured data regarding health-related behaviors (ie, general health, sleep, and degree of moderate and vigorous physical activity) and nutrition and eating habits (ie, frequency of breakfast, and number of servings of fruit and vegetables per day). These items were adapted from the US Centers for Disease Control and Prevention’s Behavioral Risk Factor Surveillance System Survey [45]. In addition, self-efficacy with regard to both physical activity and weight management were captured. Items were drawn from the Physical Activity Confidence Scale [46] and the Weight Efficacy Lifestyle Questionnaire (WEL) [47]. We used the global WEL score for this study, where high scores represent more positive beliefs toward the completion of weight management actions [48]. In addition to demographic data, the presurvey also asked questions about attitudes toward exercising at a health club with items adapted from Miller and Miller [49]. Virtual-world participants were also asked about prior experience with Second Life and, on the postsurvey, some questions about their perceptions of the environment. Lastly, virtual-world participants were given an opportunity to offer open-ended responses about the experience. Regarding our surveys, all construct reliabilities exceeded 0.70. Specific survey items and factor analytic results are available, upon request. This study was approved by Indiana University Institutional Review Board.

### Statistical Analysis

Descriptive statistics were used to describe baseline characteristics of groups. The analyses tested for group (virtual world and face-to-face) differences in baseline characteristics using a multivariate analysis of variance. We used repeated-measures multivariate analysis to detect group differences, within-participant differences (time), and interactions (group × time) in measured outcomes from baseline to postintervention. Paired *t *tests were used to identify where significant differences occurred pre- to postintervention. A 2-sample *t *test between proportions was performed to determine whether there was a significant difference between groups with respect to the percentage of weight lost. We considered differences to be significant at the *P *< .05 level and used SPSS version 18 (IBM Corporation, Somers, NY, USA) for analysis.

## Results

### Demographics

Full data sets were available for 33 virtual world and 21 face-to-face participants. Virtual-world participants (n = 5) dropped out in the first 2 weeks due to technical difficulties and personal reasons. Face-to-face participants (n = 3) dropped out midprogram, citing disinterest and personal reasons. Demographically, the virtual-world group was 76% female (25/33), with a mean age of 46.3 (SD 9.6) years; 73% (24/33) held college or advanced degrees; and 76% (25/33) had annual incomes exceeding US $75,000. All 33 reported they were novice users of Second Life (0–3 months). The face-to-face group was 95% (20/21) female, with a mean age of 37.5 (SD 10.6) years; 90% (19/21) held college or advanced degrees; and 71% (15/21) reported incomes over US $75,000. Across these data, there were no significant differences. However, regarding attitudes toward exercising at a real club, virtual-world participants reported a statistically significant (*P *= .03) higher negative attitude (mean score 3.35, SD 1.13, on a scale of 1 to 7, where 1 = strongly disagree; higher scores represent more negative attitudes) than face-to-face participants (2.69, SD 0.97).

### Change in Primary Outcome

The mean BMI of the overall sample was 32.0 (SD 6.05) kg/m^2 ^(virtual world: 33.13, SD 6.13; face-to-face: 30.21, SD 5.62). No significant baseline differences were noted between groups for weight, BMI, general health, fruit and vegetable consumption, breakfast frequency, and physical activity self-efficacy. However, significant baseline differences were observed for moderate physical activity (*P *= 0.02), vigorous physical activity (*P *= .001), sleep (*P *= .02), and WEL (*P *= .04).

Table 1 summarizes baseline and postintervention values for objective measures. No significant group × time interactions were detected; however, both groups lost a significant amount of weight (virtual world: 3.9 kg, *P *< .001; face-to-face: 2.8 kg, *P *= .002). Compared with baseline, the virtual-world group lost an average of 4.3% (range –17.3% to 3.3%), with 33% (11/33) of the participants losing a clinically significant (≥5%) amount of weight. The face-to-face group lost an average of 3.0% (range –11.0% to 2.7%), with 29% (6/21) losing a clinically significant amount. Furthermore, 15.2% (5/33) of the virtual-world and 14.3% (3/21) of the face-to-face groups lost 7% or more of their body weight. Significant differences were not detected between groups for the percentage of weight lost (*P *= .34) or the percentage of participants losing 5% or more of their baseline body weight (*P *= .39).

**Table 1:** Changes in body weight from baseline to postintervention.

Outcome variable, mean (SD)	Virtual world		Face-to-face		*P *value	
	Pre	Post	Pre	Post	Group × time interaction	Time main effect
Weight (kg)	92.1 (23.2)	88.2 (21.6)	83.9 (16.1)	81.1 (14.5)	.29	.001
Body mass index (kg/m^2^)	33.13 (6.13)	31.71 (5.51)	30.21 (5.6)	29.13 (4.96)	.39	.001

### Change in Secondary Outcomes

Table 2 summarizes baseline and postintervention values for self-reported measures of behavioral change and self-efficacy. The group × time interaction was significant for pre- to postintervention general health, moderate and vigorous physical activity, physical activity self-efficacy, fruit and vegetable consumption, and WEL. Post hoc paired *t *tests indicated significant improvements across *all *of the variables for the virtual-world group, while the face-to-face group had nonsignificant improvements in self-efficacy for physical activity and WEL, as well as for fruit and vegetable consumption. The face-to-face group reported decreases in moderate and vigorous physical activity that were nonsignificant. Lastly, we noted a significant time effect for general health and breakfast consumption. Paired-samples *t *tests indicated a significant improvement in perceptions of general health (*P *< .001) and an increase in the number of days the virtual-world group ate breakfast (*P *= .003); in contrast, there were no significant changes in these variables for the face-to-face group.

**Table 2:** Changes in health behaviors and self-efficacy from baseline to postintervention.

Outcome variable, mean (SD)	Virtual-world group		Face-to-face group		*P *value	
	Pre	Post	Pre	Post	Group × time interaction	Time main effect
Health (1 = poor to 5 = excellent)	3.0 (1.1)	3.5 (1.0)	3.2 (0.7)	3.4 (0.9)	.12	.001
Sleep (1 = <6 hours to 5 = ≥9 hours)	2.82 (0.88)	2.87 (0.78)	3.38 (0.81)	3.23 (0.89)	.30	.62
Moderate PA^a ^(no. days/week)	2.8 (2.2)	4.2 (1.7)	4.2 (2.1)	3.9 (1.9)	.006	.08
Vigorous PA (no. days/week)	1.4 (1.8)	2.5 (2.0)	3.2 (1.9)	3.0 (1.8)	.008	.04
PA self-efficacy (1 = not to 5 = extremely confident)	2.92 (0.80)	3.42 (0.80)	3.39 (0.92)	3.41 (0.80)	.04	.02
Fruit and vegetables (1 = 0 to 5 = >5/day)	2.79 (0.93)	3.37 (0.89)	2.81 (0.81)	2.90 (0.77)	.007	<.001
Breakfast (no. days/week)	6.15 (1.48)	6.78 (0.60)	6.52 (0.98)	6.67 (0.66)	.13	.02
Weight efficacy (scale 19 to 133)	80.70 (20.1)	108.7 (16.7)	92.0 (17.2)	97.3 (16.7)	<.001	<.001

^a ^Physical activity.

## Discussion

The purpose of this study was to explore the potential of a virtual-world-based weight loss program relative to weight loss, behavioral change, and self-efficacy. We used a face-to-face program that was similar in structure and content for comparative purposes. We found both groups significantly benefitted from their respective interventions in terms of weight loss and BMI reduction (see Table 1). Importantly, the average weight loss results of virtual-world participants (3.9 kg) compares favorably with studies of other short-term (12- to 16-week) programs [13,14,48,50-52]. In addition, the percentage weight lost and percentage of virtual-world participants who lost 5% or more of their body weight is also consistent with or greater than those in previous investigations [8,13,14,52]. Interestingly, virtual-world participants exhibited significant improvements in nearly all indicators (except sleep) of behavioral change, while the face-to-face participants showed *no *marked improvements in any indicator. Additionally, virtual-world participants’ self-efficacy regarding their abilities to engage in physical activity and to resist eating (WEL) both increased significantly (see Table 2), while again the face-to-face group exhbited no significant changes. In fact, the change in the WEL score (+28) for Club One Island participants was higher than has been noted in previous face-to-face and Web-based intervention studies [48,53].

As we consider these results, it is important to emphasize the tight integration of the Club One Island program and island designs. The design of both aspects allowed for motivational reinforcement, practice-oriented instruction and active learning, and social support, collectively serving to create a learning environment that fostered desired outcomes. In contrast, physical environmental limitations experienced by face-to-face participants seemed to have been less supportive of behavioral change. For example, the dynamic 3D spaces allowed virtual-world participants opportunities to test both positive and negative behaviors like navigating complex food situations, such as at a party. Our results suggest that behavioral change and increased self-efficacy were likely influenced by virtual-world participants observing their avatars engage in healthy behaviors. Virtual-world participants overwhelmingly created avatars that reflected real depictions of themselves. As they lost weight in the real world, they made changes to their avatar’s appearance to reflect this. Through this environment, participants were able—many for the first time in their lives—to have a positive experience related to physical activity, and to test both positive and negative behaviors such as navigating complex food situations. Sample comments by virtual-world participants provide evidence of avatar identification and the transfer of virtual-world behaviors to the real world, as well as the development of self-efficacy and the role of social support (for additional comments from participant interviews, see Multimedia Appendix 2):

During the workday, I remember my avatar sipping from a (3D) water bottle and I’d grab my own (real) bottle. Having the bottle and drinking animation has led directly to a change in my behavior.

Usually when I’m on the treadmill at the gym, I walk for 5 minutes and run 1 minute, which is really challenging. This last time, I pictured my avatar running and I felt like my avatar and it made me feel stonger. I ran for 2 minutes easily.

I’m integrating skills I didn’t know how to use. I usually read nutritional labels but didn’t really know what to do with the information. Now I have more confidence and know how to maintain my weight loss.

This was the best part of any class, when the other members talked about their experiences. It was just good to know other people out there are struggling with the same issues.

Overall, our results offer positive early evidence that a virtual-world-based weight loss program can be as effective as a face-to-face one relative to biometric changes. Our results also suggest that a virtual world may be a *more effective *platform to influence meaningful behavioral changes and improve self-efficacy. Nearly 70% (23/33) of participants agreed or strongly agreed that the virtual-world program worked better than other things they had tried in the past. The fact that all were novice virtual-world users suggests that deep technical skills are not a prerequisite for success.

While we do not direct compare them here, our results are also promising relative to those of prior studies of Web-based interventions. One indicator is that, despite the weekly time commitment, the dropout rate within the virtual-world program was low (15%) compared with other Web-based programs [54,55]. This suggests a greater level of engagement and less boredom with the virtual-world program, both factors that have led to attrition in Web-based programs. More specifically, a review by Lodama et al [56] suggests that the lack of face-to-face interaction and static pages are two major limitations of Web-based programs. Harvey-Berino et al [4] also found significant differences in perceived social support when comparing Web-based versus face-to-face programs, partially explaining lower and significantly different total weight lost. They concluded that “strategies to facilitate and enhance a sense of group cohesion online” are needed (p. 127). In response, recent research has focused on enhancing Web-based interventions by incorporating elements (eg individualized programs, chat sessions, forums, and more dynamic material) to increase engagement. However, to date, results of such efforts have not led to significant improvements in weight loss [54,55]. Since engagement results from a synergistic interaction between motivation and active learning [57] (p. 8), the very nature of virtual worlds may offer significant advantages over earlier technologies. As illustrated in this study, virtual worlds are inherently dynamic, and they offer significant opportunities for social interactivity and support. Importantly, through avatar self-embodiment, virtual worlds allow for a functional equivalent to face-to-face interactions, thus helping to address the above-noted shortcomings of Web-based interventions.

### Limitations and Directions

The sample in this study provided both strengths and weaknesses. Given our research focus and the interests of our industry partner, the demographics of our sample were appropriate. However, this is a possibility of a self-selection bias, as participants in both groups volunteered to participant in the study in response to a recruitment advertisement. Thus, their motivation and other possible variables may not be representative of the general population. Future research should involve a broader recruitment strategy and controlled assignment to an intervention. While our study followed past practice by using a face-to-face group for comparative purposes, the next step would also be to compare a virtual-world versus a Web-based intervention. This may allow, for example, deeper analysis of the relationship between avatar identification and outcomes.

We also focused our attention largely on outcomes to compare the effectiveness of the virtual-world and face-to-face weight loss programs. While we found positive benefits immediately following program completion, a longitudinal assessment is needed to determine whether behavioral changes are sustained over time for virtual-world participants. We have some evidence that avatar identification, virtual- to real-world behavior transfer, and social support components relate to outcomes. Yet, as with earlier studies [8], how these elements actually work together or whether relationships to outcomes are correlational or causal is not yet fully understood. Similarly, the weight loss program itself involved multiple educational components along with social support. The relative importance of each to the observed outcomes warrants further exploration. In addition, research is needed regarding the relationship between individual characteristics (eg, obesity classication, learning style, and immersive tendencies) and outcomes. For example, the degree to which one is receptive to immersive experiences during media exposure (such as a virtual world) may influence outcomes. Similarly, as a visually oriented environment, a virtual world may be more attractive to individuals with a better spatial sense and who prefer learning through images and objects, rather than text.

Lastly, from a human–computer interaction design perspective, a deeper understanding of how design elements (eg, environmental or avatar realism) interact with individual characteristics (eg, a preferred representational system) is essential to advancing the adoption of virtual worlds. Moreover, while a virtual presence is considered central to the utility of a virtual world, attempts to design spaces to maximize presence are premature without solid evidence that it relates to desired outcomes. In this study, we also explored the efficacy of a specific virtual-world platform (Second Life). Further work should examine alternative platforms possessing varied sociotechnical capabilities.

In sum, further empirical testing in both controlled experiments and field studies would help to develop a richer understanding of the value of virtual-world-based interventions to address obesity and overcome the challenges of existing approaches.

### Conclusion

The rising rates, high prevalence, and adverse consequences of obesity call for the development and testing of innovative approaches that address the cited barriers and bring needed help to those most affected. While more research is needed into their use in medical and health contexts [31], as shown here, virtual worlds may offer an environment within which participants can engage in experiential learning and simulate what-if scenarios without serious repercussions. The affordances of simulated 3D experiences, anonymity, embodied personal representation in the form of an avatar, and rich social interaction constitute the potential for a virtual world to have a strong effect as a weight loss intervention platform. While use and research is in its infancy, there are encouraging signs regarding application to a variety of medical, health, educational, and other contexts. We hope that the ideas and findings offered here contribute to this growing area.

## Multimedia Appendix 1

Club One Island Virtual Spaces and Activities.

## Multimedia Appendix 2

Participant Interview.

## Abbreviations

3D: 3-dimensional
BMI: body mass index
WEL: Weight Efficacy Lifestyle Questionnaire
